# Caloric restriction reprograms skeletal muscle molecular pathways in non-human primates: potential relevance to human aging biology

**DOI:** 10.1186/s13395-026-00422-9

**Published:** 2026-05-08

**Authors:** Jayanta Kumar Das, Nirad Banskota, Stefano Donega, Nader Shehadeh, Yulan Piao, Nathan Price, Julie A. Mattison, Julián Candia, Rafael de Cabo, Luigi Ferrucci

**Affiliations:** 1 Longitudinal Studies Section, Translational Gerontology Branch, National Institute on Aging, National Institutes of Health, Baltimore, MD 21224 USA; 2 Computational Biology and Genomics Core, National Institute on Aging, National Institutes of Health, Baltimore, MD 21224 USA; 3 Intramural Research Program, National Institute on Aging, National Institutes of Health, 251 Bayview Blvd., Suite 101, Baltimore, MD 21224 USA; 4 RNA Regulation Section, Lab of Genetics and Genomics, National Institute on Aging, National Institutes of Health, Baltimore, MD 21224 USA; 5 Experimental Gerontology Section, Translational Gerontology Branch, National Institute on Aging, National Institutes of Health, Baltimore, MD 21224 USA

**Keywords:** Caloric restriction, Non-human primates, Muscle, Gene expression, RNA, Splicing

## Abstract

**Background:**

Caloric restriction (CR), achieved by reducing energy intake without malnutrition, has been shown to preserve muscle function and delay age-related declines in strength and mobility by modulating key metabolic and molecular pathways involved in muscle maintenance. While most initial research on CR was done in rodents, non-human primates (NHPs) offer a higher translatable animal model for understanding CR effects due to their close genetic, physiological and cognitive similarities to humans.

**Methods:**

In this cross-sectional study, we investigated skeletal muscle gene expression changes induced by 30% CR in skeletal muscle in rhesus monkeys (*n* = 18 CR, *n* = 18 control). We performed high-depth RNA sequencing to profile gene expression and alternative splicing variants and identify pathways linked to aging, regeneration/degeneration, and energy metabolism.

**Results:**

Transcriptomic profiling revealed widespread gene expression differences between CR animals compared to controls. Genes that were overexpressed were mainly involved in pathways related to energy metabolism, mitochondrial function, signaling, and oxidative stress response. Conversely, underexpressed genes were connected to immune response, extracellular matrix organization, apoptosis, and ribosomal RNA processing. Further, we identify alternative splicing as a major mechanism by which CR modulates genes involved in muscle function, metabolism, and aging.

**Conclusions:**

Caloric restriction preserves skeletal muscle by enhancing metabolism, limiting degeneration and inflammation, and engaging conserved mechanisms across species.

**Supplementary Information:**

The online version contains supplementary material available at 10.1186/s13395-026-00422-9.

## Introduction

Chronic calorie restriction (CR) promotes the preservation of muscle mass and function despite weight loss in rodents models, as it promotes a reduction in fat mass without significant muscle loss [1]. When calorie intake is gradually decreased and adequate protein and nutrients are maintained, the body prioritizes burning fat for energy, protecting lean muscle [2–4]. Preserving muscle is essential because muscle tissue is metabolically active and increases the resting metabolic rate, supporting long-term weight control [5, 6]. Additionally, maintaining muscle mass is crucial for strength, mobility, and overall metabolic health, making CR an effective method for sustainable fitness and overall health and well-being.

Skeletal muscle plays a central role in resting energy expenditure due to its substantial mass and metabolic activity [7]. Multiple studies across species- including mice, rats, non-human primates (NHPs), and humans have shown that CR helps preserve skeletal muscle mass and improve muscle strength and function [6, 8–17]. In our previous study performed on muscle biopsies from humans involved in a randomized controlled clinical trial of CR, CR has been shown to beneficially reshape muscle transcriptomic profiles, improving muscle function and glucose homeostasis [18]. At the molecular level, CR leads to a reduction in oxidative damage to DNA and proteins [14, 15], increase in mitochondrial biogenesis [19, 20], and improvements in mitochondrial function and energy metabolism [19, 21]. CR has also been linked to improved cognitive function and a reduction in neuroinflammation [22–24]. CR promotes autophagy and cellular quality-control mechanisms through the upregulation of key molecular chaperones and autophagy regulators, leading to improvements in proteostasis and the removal of dysfunctional organelles [25, 26]. These adaptations improve aerobic capacity, reduce muscle fiber loss, and mitochondrial abnormalities in most models [5, 8, 17, 27]. In parallel, CR also improves metabolic health of skeletal muscle through enhancing insulin sensitivity, promoting lipid turnover, and reducing the production of harmful metabolites [13, 28–30].

Transcriptomic and physiological analyses in CR studies consistently reveal the upregulation of genes involved in oxidative phosphorylation, mitochondrial maintenance, and autophagy—hallmarks of improved cellular resilience [18, 31]. At the same time, CR downregulates pro-inflammatory and aging-associated signaling pathways, including mTOR signaling, cytokine production, NF-κB, and its upstream regulator PI3K/AKT, contributing to a more youthful transcriptional landscape [6, 25, 32–35]. Notably, pharmacological mTORC1 inhibition by rapamycin has been shown to counteract skeletal muscle aging through mechanisms that are partially overlapping yet distinct from CR, suggesting that multiple nutrient-sensing pathways converge to modulate muscle health [36, 37]. In rhesus monkeys, CR also reverses age-related alterations in skeletal muscle microRNA expression, further supporting its role in delaying molecular aging [38]. While CR generally improves insulin sensitivity and lipid turnover in muscle [28, 35], some studies have noted variability in metabolic responses, particularly regarding glycogen metabolism and insulin action in primate models [39, 40]. Moreover, comparisons between CR and exercise suggest differential effects on mitochondrial enzyme activity and lipid metabolism, especially in older adults [41]. Together, these findings highlight CR’s complex role in supporting muscle structure and function, delaying sarcopenia, and enhancing metabolic health and longevity. However, despite strong associative evidence, the exact causal mechanisms through which CR influences skeletal muscle aging are still not fully understood. In particular, transcriptomic insights from NHPs remain limited, leaving an important gap in our cross-species understanding of CR-driven muscle adaptations.

NHPs and humans share strong physiological and genetic similarities. Our previous study [42, 43] demonstrated that the health benefits of CR are conserved in NHPs, supporting the translatability of CR mechanisms to human health. While CR has been shown to improve metabolic health and extend lifespan across species, the tissue-specific molecular pathways that mediate these effects, particularly in skeletal muscle, are not well characterized. Muscles play a central role in systemic metabolism, mobility, and aging, making it a critical tissue for understanding CR’s impact at the molecular level [44, 45]. However, muscle-specific transcriptomic investigations in NHPs under CR remain limited. Studying these responses can help uncover key regulatory pathways and identify potential therapeutic targets for age-related muscle decline and metabolic disorders in humans.

This study focuses on a subset of muscle transcriptomic data from NHPs included in our larger CR study [43]. We hypothesize that CR causes specific changes in muscle transcriptomic profiles in NHPs compared to control groups. Specifically, we suggest that CR influences key metabolic and regulatory pathways that control muscle function, adaptation, and overall health. By identifying these gene expressions and alternative splicing changes, this study aims to clarify the molecular mechanisms through which CR affects muscle maintenance and lifespan. Expanding investigations into NHP models will improve cross-species understanding of CR’s impact on muscle physiology, providing essential translational insights that connect mechanistic findings from rodents to humans and guide strategies for enhancing muscle health and longevity.

## Methods

### Animal study and sample collection

A total of forty male and female rhesus monkeys between the ages of 20 and 42 years (average = 30.1), were selected based on tissue quality and availability. These animals were part of a larger, longitudinal study conducted at the National Institute on Aging (NIA) to investigate the effects of long-term CR on health and longevity [42, 43]. Procedures were approved by the NIA Intramural Research Program’s Institutional Animal Care and Use Committee. CR monkeys (*n* = 20) consumed about 30% fewer calories than their age-, sex-, and body weight-matched controls (*n* = 20) (450–600 vs. 600–800 kcal daily) throughout the lifespan of the study, as previously described [43]. Animals were provided with a specially formulated diet (NIA-1-87) that included a 40% surplus of vitamins and minerals to compensate for reduced intake in CR monkeys. Monkeys were humanely euthanized when clinically indicated, at which time tissues were harvested. The vastus lateralis was removed and flash frozen in liquid nitrogen in individual aliquots. Tissues were stored at -80° C until processing for RNA-sequencing.

### RNA extraction and mRNA sequencing

RNA was collected from flash frozen vastus lateralis skeletal muscle biopsies (~ 50–100 mg) using RNeasy Fibrous Tissue Mini Kit protocol (Qiagen, Cat. No. 74704). To automate the process, we used QIAcube Connect (Qiagen, Cat. No. 9002864). We collected ca. 2,543 ng RNA per sample with average concentration of 185 ng/uL. RNA was sequenced using short-reads Illumina NovaSeq 6000 system with paired-end reads. The sequenced RNA samples yielded 426 to 622 million pass filter reads with more than 90% of bases above the quality score of Q30. Four samples with degraded RNA (RIN < 3) were excluded, resulting in thirty-six high-quality samples with equal male to female ratio for downstream analysis.

### Processing of raw RNA-seq data

The quality of reads in fastq RNA-Seq files were initially assessed using FastQC tool (version 0.12.1; https://www.bioinformatics.babraham.ac.uk/projects/fastqc/). Reads were trimmed using bbduk (from bbtools package; https://jgi.doe.g.ov/data-and-tools/software-tools/bbtools/ [46]). Following trimming, cleaned reads were examined one more time using FastQC. Next, cleaned FASTQ files were aligned to the *Macaca mulatta* reference genome (assembly Mmul_10) using STAR (version 2.7.10b) [47], a splice-aware aligner optimized for high-throughput RNA-seq data. Genome indices were generated with the corresponding Ensembl annotation file (Mmul_10.107.gtf), enabling accurate mapping of splice junctions and transcript structures. The STAR aligner was run with the—quantmode TranscriptomeSam parameter to also generate transcriptome BAM files and was used for RSEM (version 1.3.3) analysis. Genome BAM files were sorted and indexed using samtools (version 1.21). Finally, genome BAM files were used as input for featureCounts from the Rsubread package (version 2.0.1) [48], a suitable program for counting reads for various genomic features such as genes.

### Differential gene expression analysis

Raw count data were input into the DESeq2 (version 1.44.0) model [49] for differential expression analysis between CR and control groups, adjusting for diet, sex, age, weight and batch effects. Genes with an unadjusted p-value < 0.01 were considered significantly differentially expressed. The most significant genes associated with biological mechanisms influenced by CR were selected for further functional characterization through literature review and GeneCards database [50].

### Functional enrichment and pathway analysis

Following established protocols for pathway enrichment analysis of ranked gene lists encompassing a large portion of the genome, we conducted PreRank Gene Set Enrichment Analysis (GSEAPreranked), a threshold-free method that evaluates all measured genes without prior filtering [51]. Gene ranks were calculated using the formula r = -log10(p-value) * sign(beta), where beta represents the log₂ fold change between CR and control, integrating both the magnitude and direction of differential expression. Enrichment analysis was conducted using GSEA (version 4.3.2), referencing gene sets from the Molecular Signatures Database (MSigDB version 7.4) [52]. We focused on two curated collections: Canonical Pathways (including Reactome, 1,615 gene sets) and Hallmark (50 gene sets). Pathways with false discovery rate (FDR) adjusted p-values < 0.05 were considered significantly enriched.

### Differential alternative splicing analysis

Transcript abundance was quantified using RSEM, which performs full transcript alignments and offers improved accuracy in handling multi-mapping reads and closely related isoforms (Li & Dewey, 2011; see Sect.  4). Abundances were reported as Transcripts Per Million (TPM) and then rescaled into count-like values using the “dtuScaledTPM” option, which adjusts TPMs in a manner that accounts for differential transcript usage (DTU). These transcript-level counts were subsequently analyzed with DRIMSeq, a statistical framework that models relative isoform proportions using a Dirichlet–multinomial distribution and tests for DTU between CR and Control groups. Prior to statistical testing, we applied DRIMSeq’s filtering procedure to remove lowly expressed or unstable features, requiring transcripts to be expressed in at least ~ 20% of samples with ≥ 20 counts and a minimum proportion > 0.03 in those samples, and genes to be expressed in at least ~ 40% of samples with ≥ 40 counts. After filtering, 21,549 transcripts from 7439 genes remained for downstream analysis.

### Software and data visualization

All statistical analyses were conducted in R (version 4.2) on the x86_64-w64-mingw32 platform. Data visualizations—including volcano plots, box plots, and bar plots—were generated using the ggplot2 package (version 3.5.1) within the R environment. Primary analysis steps (e.g., read mapping, expression quantification, differential analysis, and enrichment) were carried out using standard bioinformatics tools as detailed in the sections above.

## Results and discussion

### Characteristics of study population and phenotypic comparison between calorie restriction and control groups

**Fig. 1:**
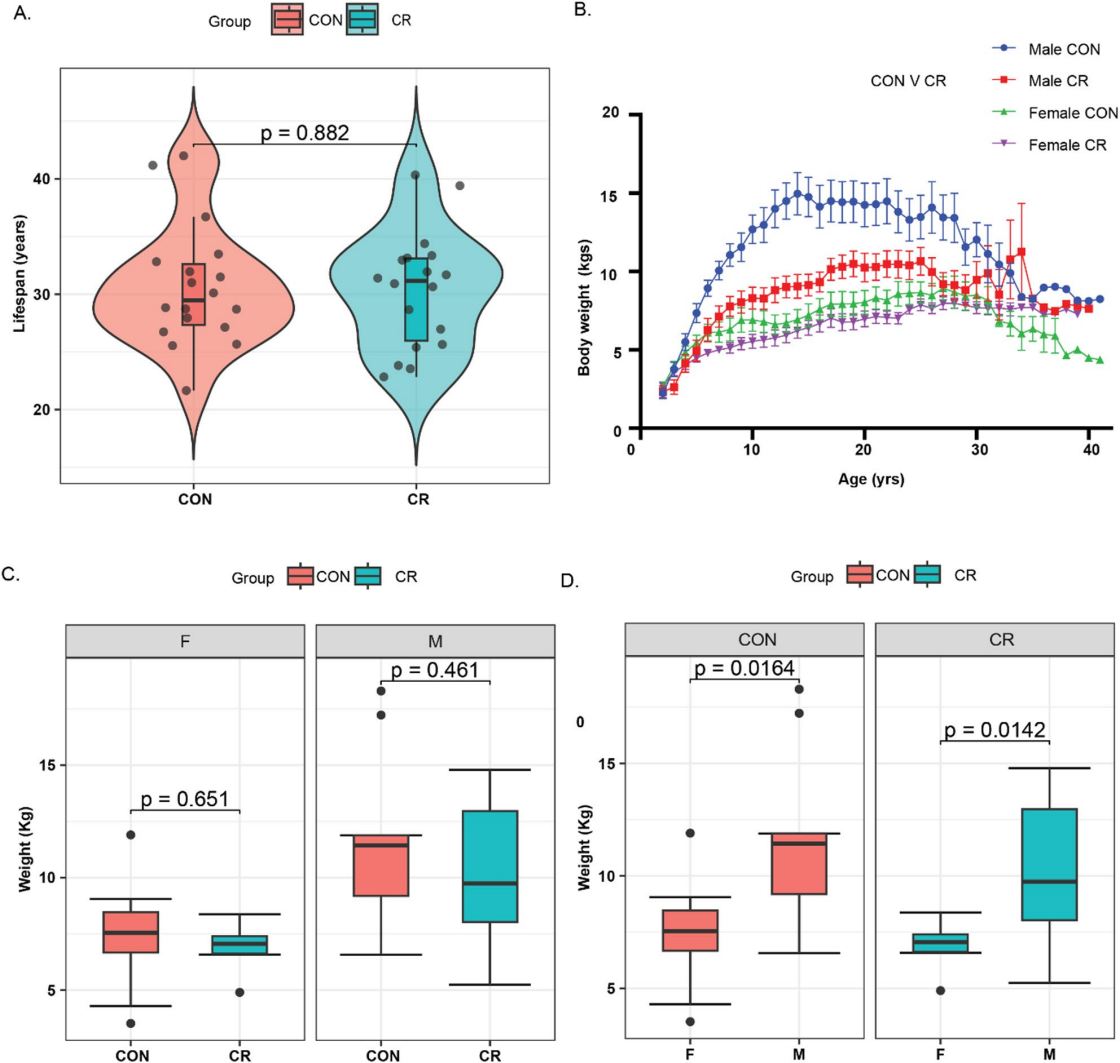
Distribution of lifespan (**A**) and body weight (**B**–**D**) for calorie restricted (CR) and control (CON) groups, presented for combined and sex-separated samples (M-Male, F-Female). **A** Violin plot illustrating the lifespan distribution (age at death) in CR and CON groups. **B** Line plot depicting body weight trajectories across the lifespan (age at different timepoints) for CR and CON groups. **C** Box plot comparing body weight (after death) distributions between CR and CON groups, separated by sex. **D** Box plot showing sex-specific body weight (collected after death) distributions within each group of CR and CON

Analysis of this focused on a sub-cohort of thirty-six (after excluding four animal due to their degraded RNA as mentioned in method) rhesus monkeys (*Macaca mulatta*) selected from a larger longitudinal CR study cohort previously described in detail [42]. Vastus lateralis muscle biopsies were collected after death from 18 CR and 18 control animals, with balanced representation of males and females in each group. The average lifespan of monkeys in the CR group was 30.0 ± 5.29 years (range: 20.6–40.3), while that of the control group was 30.3 ± 5.4 years (range: 20.7–42.0), with no significant difference between groups (Fig. 1A). In terms of body weight, the CR group maintained a significantly lower body weight than controls until approximately age 30 (Fig. 1B). At the time of death, the CR group had a lower mean weight (8.45 ± 2.83 kg) compared to the control group (9.31 ± 3.76 kg), but the difference between CR and controls was not statistically significant (Fig. 1C). When analyzed by sex, males weighed significantly more than females in both CR and control groups (Fig. 1D). These findings in the sub-cohort enrolled in this study mirror the pattern reported in the full cohort [42]. These comparable weight distributions further ensure the reliability of our comparisons by minimizing potential confounding effects related to body mass differences.

### Differential gene expressions in skeletal muscle reveals CR-induced transcriptomic remodeling

**Fig. 2:**
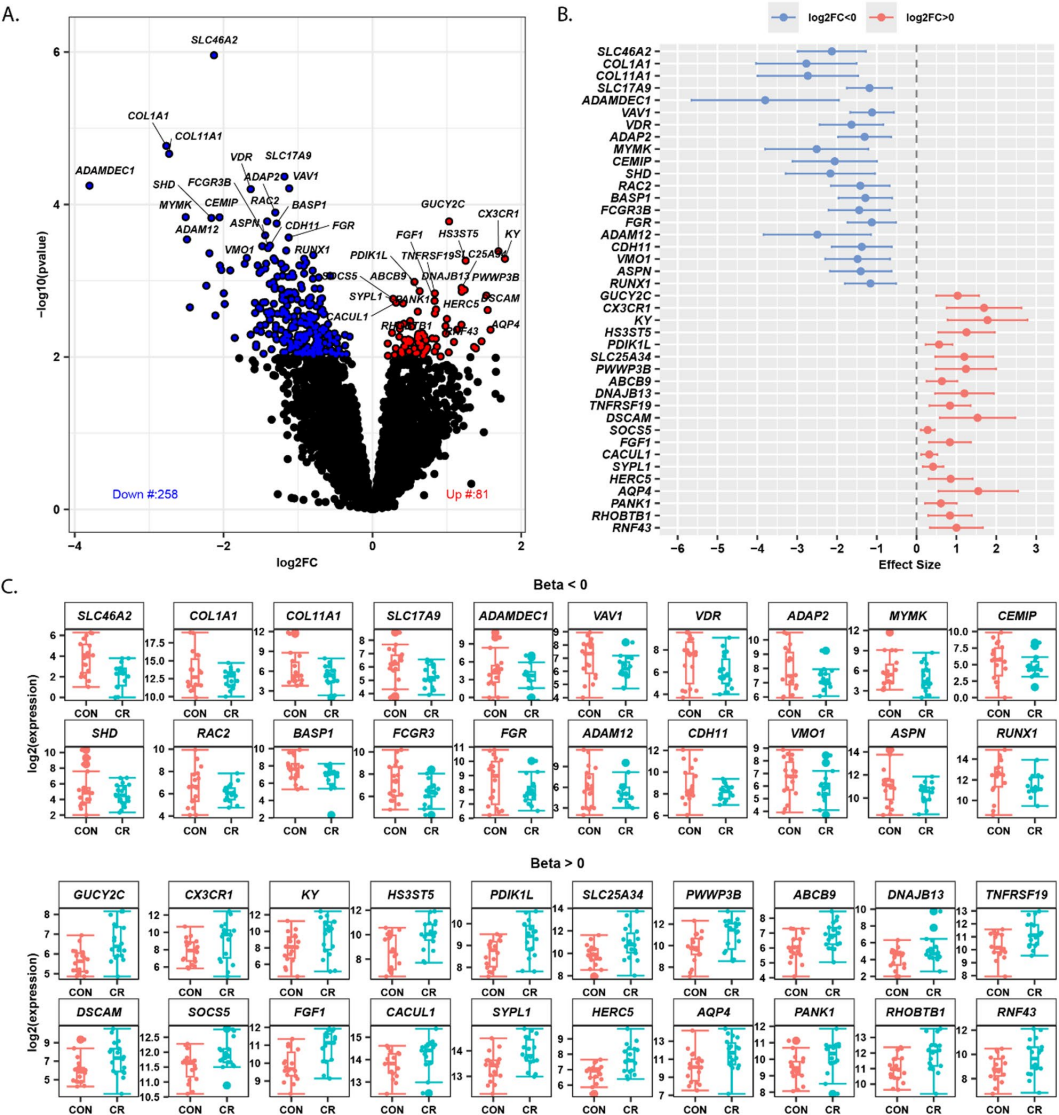
Differential gene expression analysis between calorie restriction (CR) and control (CON) groups. **A** Volcano plot displaying log2 fold changes versus statistical significance (-log10 p-value) for all detected transcripts. Red points indicate significantly upregulated genes in CR (positive β, *p* < 0.01), blue points represent significantly downregulated genes in CR (negative β, *p* < 0.01), and gray points show non-significant genes. **B** Forest plot of the top 40 differentially expressed transcripts ranked by p-value, displaying β coefficients with 95% confidence intervals for the 20 most significantly upregulated (β > 0, top panel) and 20 most significantly downregulated (β < 0, bottom panel) genes in CR versus CON. **C** Box plots showing normalized expression levels (log2 transformed) for the same top 40 differentially expressed genes, comparing calorie restriction (CR) and control (CON) groups. Each panel displays 20 genes arranged in two rows of 10 genes each, ordered by statistical significance (lowest p-values first)

We performed differential gene expression analysis between the CR and control groups using the DESeq2 statistical framework [49](see Methods). When applying FDR-adjusted p value < 0.05, only a small number of genes were significant; therefore, we applied a threshold of *p* < 0.01 to capture broader transcriptional patterns. Using a significance threshold of *p* < 0.01, we identified 339 significantly differentially expressed genes. Among these, 81 genes were overexpressed and 258 were underexpressed in the CR group relative to controls, as shown in the volcano plot (Fig. 2A). Notably, the CR group showed a greater number of underexpressed than overexpressed genes, reflecting an overall trend toward reduced transcript levels in skeletal muscle. To further characterize these changes, we examined the 20 most significantly upregulated and downregulated genes, ranked by the lowest p-values, which exhibited log₂ fold changes ranging from − 3.8 (underexpressed) to + 1.69 (overexpressed), reflecting moderate to large expression changes in both directions. These genes are presented in a forest plot (Fig. 2B), displaying their log2 fold changes and confidence intervals to illustrate both the magnitude and directionality of expression changes. Notably, the expression patterns of these top genes clearly distinguish CR from the control groups, highlighting the consistency and potential biological relevance of CR-induced skeletal muscle transcriptomic remodeling (Fig. 2C). To understand the functional significance of these differentially expressed genes, we analyzed their biological roles particularly in muscle physiology, aging processes. Some of these differentially expressed genes suggest mechanisms by which CR might affect muscle health. For example, For example, the reduced expression of *COL1A1* and *COL11A1* under CR is consistent with prior evidence that CR downregulates collagen-associated genes in skeletal muscle [9], and aligns with broader evidence implicating collagen regulation in fibrotic and metabolic stress contexts [53], and is further supported by reports that CR decreases ECM remodeling gene expression in adipose tissue [54]. The overexpression of the vitamin D receptor (VDR)—a nuclear receptor that regulates gene expression in response to vitamin D—is consistent with previous findings showing that caloric restriction (CR) influences skeletal muscle remodeling [55] and plays a crucial role in aging and age-related diseases [56, 57]). Other notable genes differentially expressed by CR included *MYMK* (myoblast function, muscle development and regeneration [58, 59]) and *RAC2* (involved in oxidative stress and immune signaling [60, 61]). CR reduces oxidative stress and inflammation [62] and it has been suggested that part of the anti-aging effect of CR is mediated by *RAC2* [63]. The under expression of *ADAM12* may affect muscle health by suppressing muscle regeneration [64, 65]). Key overexpressed genes were *KY* (influences muscle energy metabolism [66]), *CX3CR1* (anti-inflammatory effects in aging; skeletal muscle injury repair by regulating macrophage phagocytosis function; and trophic growth factor production [67]), *FGF1* (involved in muscle development and regeneration [68] and upregulated in response to dietary stress [69]), *AQP4* (regulates waterflow of myofibers, maintains homeostasis in skeletal muscle [70–72], plays important role in Alzheimer diseases during intermittent fasting [72]). *PANK1* (Regulator of CoA synthesis; critical for fatty acid metabolism and mitochondrial function, upregulated high-fat diets (HFDs) and during dietary restriction [73]). Beyond the top twenty genes, we also examined a broader set of up to 50 overexpressed and 50 underexpressed genes, some of which are associated with muscle physiology, aging, and responses to caloric restriction, as summarized in Table S1.

Next to determine whether the transcriptional response to CR differed between males and females, we fitted a DESeq2 model including a sex (Male, Female) × diet (CR, CON) interaction term while adjusting for age, weight, and batch. Examination of the top 30 interaction genes ranked by p value revealed diverse functional categories. Genes showing relatively stronger CR-associated effects in males included transcriptional regulators (SALL3, SOX3, IRX5), cilia/cytoskeletal genes (FOXJ1, DNAI1), and signaling factors (RASD1, TLR9), whereas genes with comparatively greater effects in females included receptor and developmental genes such as P2RY13, ANGPTL1, and MAB21L2 (Fig. S1A, Table S2). Sex-stratified analyses further indicated that CR vs. CON induced partially overlapping but distinct transcriptional changes in males and females (Fig. S1B–D, Table S2). In males, CR was primarily associated with downregulation of extracellular matrix genes (e.g., COL1A1, COL11A1, ADAM12), whereas females showed modulation of signaling and regulatory genes (e.g., AQP4, FOXL3, MEF2C, CX3CR1). These results confirm previous evidence that the biological response to CR is different in different sexes [74–76].

### Gene set enrichment analysis identifies key pathways modulated by caloric restriction in skeletal muscle

**Fig. 3:**
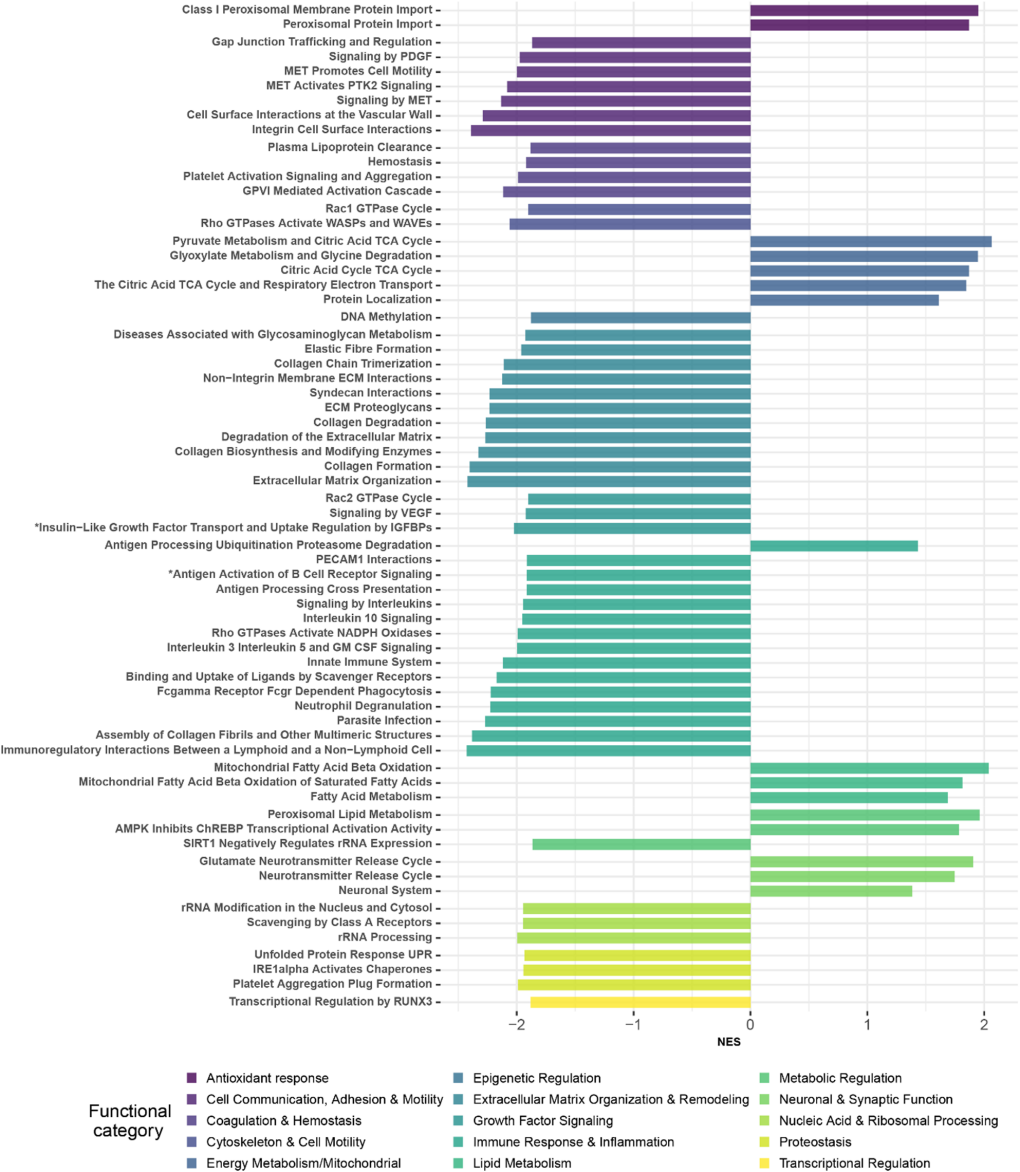
Significantly enriched reactome pathways (FDR-adjusted *p* < 0.05) identified by pre-ranked gene set enrichment analysis (GSEAPreranked). The figure displays all 16 significantly upregulated pathways and the top 50 downregulated pathways ranked by FDR-adjusted p-values. The complete list of enriched pathways, including full pathway names (indicated by *), is provided in Table S3

We then performed pre-ranked gene set enrichment (GSEAPreranked) analysis to identify top enrichments gene sets across diverse biological processes and pathways, focusing on two major reference gene set collections: Hallmarks and Reactome (Methods). Withing the Reactome collection, 149 pathways were significantly enriched (FDR-adjusted p-value < 0.05); with 16 pathways upregulated and 133 downregulated in CR (Table S3). Similarly, in the Hallmark collection, 27 pathways were significantly enriched (FDR-adjusted p-value < 0.05) including 8 upregulated and 19 downregulated in CR (Table S3). For visualization, we display all 16 significantly upregulated pathways and the top 50 downregulated pathways ranked by FDR-adjusted p-values (Fig. 3). Both Hallmark and Reactome enrichments were considered in the overall interpretation skeletal muscle transcriptional changes under CR. The upregulated pathways were predominantly associated with biological function categories such as metabolism and lipid processes, mitochondrial function, neuronal activity, antioxidant response, cell communication, adhesion and motility. In contrast, the downregulated pathways were mainly related to inflammatory responses, collagen formation, ECM organization, Coagulation and hemostasis, and Nucleic and ribosomal RNA processing response, proteostasis, oxidative stress response. Notably, several key findings from this study are consistent with results observed in other model organisms and parallel insights from our recent human studies in muscle samples [6]. This convergence across species strengthens the growing body of evidence that CR engages conserved molecular pathways that contribute to the maintenance of skeletal muscle health. Moreover, these pathways are increasingly recognized for their roles in promoting longevity and mitigating age-related functional decline. Collectively, our findings underscore the translational relevance of CR and highlight its potential as a strategy for fostering healthy aging through the preservation of muscle integrity. Some of these key pathways are discussed below.

#### Metabolic processes

Our analysis revealed an upregulation of several metabolic pathways, including lipid metabolism (Fatty acid and bile metabolism) (Fig. 3, Table S3). Although CR generally leads to a reduction in metabolic rate, certain pathways and biological processes that enhance energy efficiency, maintain cellular function, and promote survival tend to be upregulated [77]. Fatty acids serve as key source of energy generation, while bile acids facilitate lipid digestion and absorption, suggesting that CR triggers a conserved cellular response to optimize energy utilization under reduced caloric intake. This metabolic adaptation has been consistently observed across various CR studies in mice [78–81], rats [82], monkey [83] and humans [35]. Such cross-species consistency underscores the potential of CR-induced metabolic reprogramming as a universal strategy to delay aging and mitigate age-related decline.

#### Mitochondrial function and energy metabolism

During CR, several key mitochondrial pathways are upregulated, including mitochondrial fatty acid beta-oxidation of saturated fatty acids, pyruvate metabolism, the citric acid (TCA) cycle, and respiratory electron transport (Fig. 3, Table S3). These pathways function synergistically to enhance cellular energy production and support muscle mitochondrial function [20, 21, 84]. Increased mitochondrial fatty acid oxidation provides a crucial energy source [79], while the TCA cycle and electron transport chain maximize ATP production efficiency and mitigate oxidative stress. Additionally, pyruvate, a glycolysis byproduct, serves as a pivotal metabolic intermediate—either entering the TCA cycle via conversion to acetyl-CoA for energy generation or fueling gluconeogenesis for glucose synthesis [85]. Furthermore, glyoxylate metabolism and glycine degradation contribute to metabolic balance and adaptability [86]. Collectively, these enhanced mitochondrial and metabolic processes play a vital role in sustaining cellular function and resilience, particularly during aging.

#### Neuronal activities and neuromuscular signaling

Aging is linked to cognitive decline, but CR has been shown to mitigate these impairments. Here, CR upregulated several neuronal activity-related pathways (Fig. 3, Table S3), which may reflect either brain-related benefits or peripheral nervous system activity, particularly in maintaining neuromuscular integrity. Emerging studies suggest a muscle-to-brain signaling axis, where activation of muscle pathways enhances brain neurotrophic and synaptic signaling, improving cognition in models of neurodegeneration [87]. Furthermore, studies have shown that CR and/or intermittent fasting enhance brain health by promoting neuronal activity and providing neuroprotective effects. These include regulation of corticosterone levels and regional GABAergic signaling in the brain [22], reduction of memory impairment and neuroinflammation in obese aged rats [23], and elevated levels of brain-derived neurotrophic factor (BDNF) [88]. Additional mechanisms include modulation of the neuro-immune system modulating neuro-immune system [24] and changes in microRNA (miRNA) expression profiles in aging brains [89]. Although the role of dietary nitrate in enhancing CR’s effects on brain health remains under investigation [90], our findings suggest that CR may activate neuronal pathways in peripheral tissues such as muscle, which could contribute both to improved neuromuscular integrity and potentially to systemic effects that benefit brain function. Further literature review and experimental validation are needed to clarify whether these transcriptional signatures in muscle reflect direct benefits to the brain, the peripheral nervous system, or both.

#### Antioxidant defense and peroxisomal response

Several peroxisomal protein related pathways are upregulated with CR (Fig. 3, Table S3). Peroxisomal protein import is essential for antioxidant defense, and disruptions in this process can lead to oxidative stress and metabolic dysregulation [91, 92]. Under CR, the upregulation of peroxisomal protein import machinery supports improved antioxidant responses, enhancing cellular resilience and longevity. Moreover, CR cells activate various adaptive mechanisms to maintain homeostasis and protect against oxidative stress. One key response is the upregulation of antioxidant pathways, which helps to mitigate oxidative damage and enhance cellular resilience [93, 94].

#### Immune system and inflammatory response

We identified key downregulated pathways related to inflammatory and immune responses (Fig. 3, Table S3). The suppression of these inflammatory pathways suggests a reduction in inflammatory activity potentially mediating some of the beneficial effects of CR [25, 95, 96]. Notably, the signaling pathway involving interleukin-6 (IL-6), Janus kinase (JAK), and signal transducer and activator of transcription 3 (STAT3) plays a critical role in inflammatory responses and various cellular processes [97, 98]. While excessive energy intake and adiposity have been shown to induce systemic inflammation, a growing body of clinical and experimental evidence demonstrates that CR—when not leading to malnutrition—can delay aging and exert potent anti-inflammatory effects across different pathological conditions [79, 99]. Recent animal studies suggest that CR can inhibit colon tumor development and progression by reducing cellular proliferation and inflammation [100–106]. Given that excessive calorie intake and adiposity contribute to systemic inflammation, CR, when practiced without malnutrition, emerges as a promising strategy for addressing inflammation and related inflammatory diseases [95].

#### Extracellular matrix organization and collagen remodeling

In our study, we observed downregulation of ECM- and collagen-associated pathways under calorie restriction (Fig. 2, Table S3), suggesting a potential alteration in ECM remodeling processes that typically occur with aging. Recent work in *C. elegans* has demonstrated that adequate ECM and collagen remodeling is critical to healthy longevity, with pro-longevity interventions delaying age-associated collagen stiffening [107]. This confirms previous studies which have highlighted the impact of CR on ECM and collagen-associated genes and pathways. For example, CR structurally and functionally affected the skin to reduce collagen glycation [108]. At first glance, this observation could appear inconsistent with the notion that CR preserves collagen turnover during aging. However, increased collagen content in aging skeletal muscle does not necessarily reflect enhanced physiological turnover; rather, it is frequently associated with maladaptive fibrotic remodeling characterized by excessive deposition, reduced degradative capacity, and increased cross-linking, ultimately leading to matrix stiffening and impaired tissue adaptability. In this context, the reduced expression of ECM-related genes under CR may reflect attenuation of age-associated profibrotic signaling rather than suppression of healthy matrix maintenance. Thus, our findings are more consistent with CR limiting pathological ECM expansion and preserving matrix homeostasis, thereby maintaining structural integrity without promoting fibrosis. This interpretation aligns with the broader concept that CR acts to restrain age-related remodeling programs that compromise tissue resilience, rather than simply reducing anabolic processes.

#### Apoptosis and stress response regulation

Our study highlighted the downregulation of apoptosis, cell death, and stress response-related pathways in CR (Hallmark collection in Table S3). Apoptosis is generally upregulated during CR, contributing to its potential anti-cancer and anti-aging effects by eliminating damaged or unnecessary cells [109, 110]. However, several studies have shown that CR can also suppress apoptosis in certain contexts. For example, CR suppressed apoptotic cell death in the mammalian cochlea, potentially preventing presbycusis [111], and reverses age-related increases in kidney apoptosis and oxidative stress, highlighting its anti-aging and protective effects [112]. CR enhances the removal of damaged or dysfunctional muscle cells through apoptosis. Mitochondria, which are critical for energy production, accumulate damage over time, particularly in muscle tissue due to its high energy demands [86]. CR may help selectively induce apoptosis in cells with dysfunctional mitochondria, thereby maintaining muscle tissue quality and function by eliminating compromised cells.

#### Ribosomal RNA processing

Ribosomal RNA (rRNA) processing pathways were downregulated under CR (Fig. 3, Table S3), suggesting altered regulation of ribosome biogenesis–related pathways. It is important to note that changes in expression of rRNA processing genes do not directly implies rRNA reduced abundance or protein synthesis rates, which were not measured in the present study. Reduced ribosome biogenesis has been proposed as a feature of CR and longevity-associated programs in various model systems [2, 113]. Notably, aging is associated with increased DNA methylation at the ribosomal DNA locus, which suppresses rRNA transcription. CR may partially reverse this epigenetic modification, further contributing to the reduction in rRNA production and enhancing its positive effects on cellular function [114, 115]. However, whether CR modulates these mechanisms or functionally impacts ribosome biogenesis in skeletal muscle cannot be determined from the current transcriptomic data.

### Differential transcript usage of alternative splicing changes in response to calorie restriction

After characterizing differential gene expression, we tested the hypothesis that CR modulate the spliceosome leading to the emergence of splicing isoforms. This is consistent with the accumulating evidence that CR is a form of stress that trigger response mechanisms that are in part mediated by RNA isoform diversity. Leveraging the high sequencing depth of our paired RNA-Seq dataset, we quantified transcript abundances using RSEM and assessed differential transcript usage with DRIMSeq (see Methods) under a stringent significance threshold (*p* < 0.01). From ~ 21,549 splicing variants tested across 7439 genes; we identified 389 splicing variants differentially expressed between CR and controls across 311 genes. Notably, 78 genes produced two or more significant variants, indicating potential regulatory hotspots of alternative splicing.

**Fig. 4:**
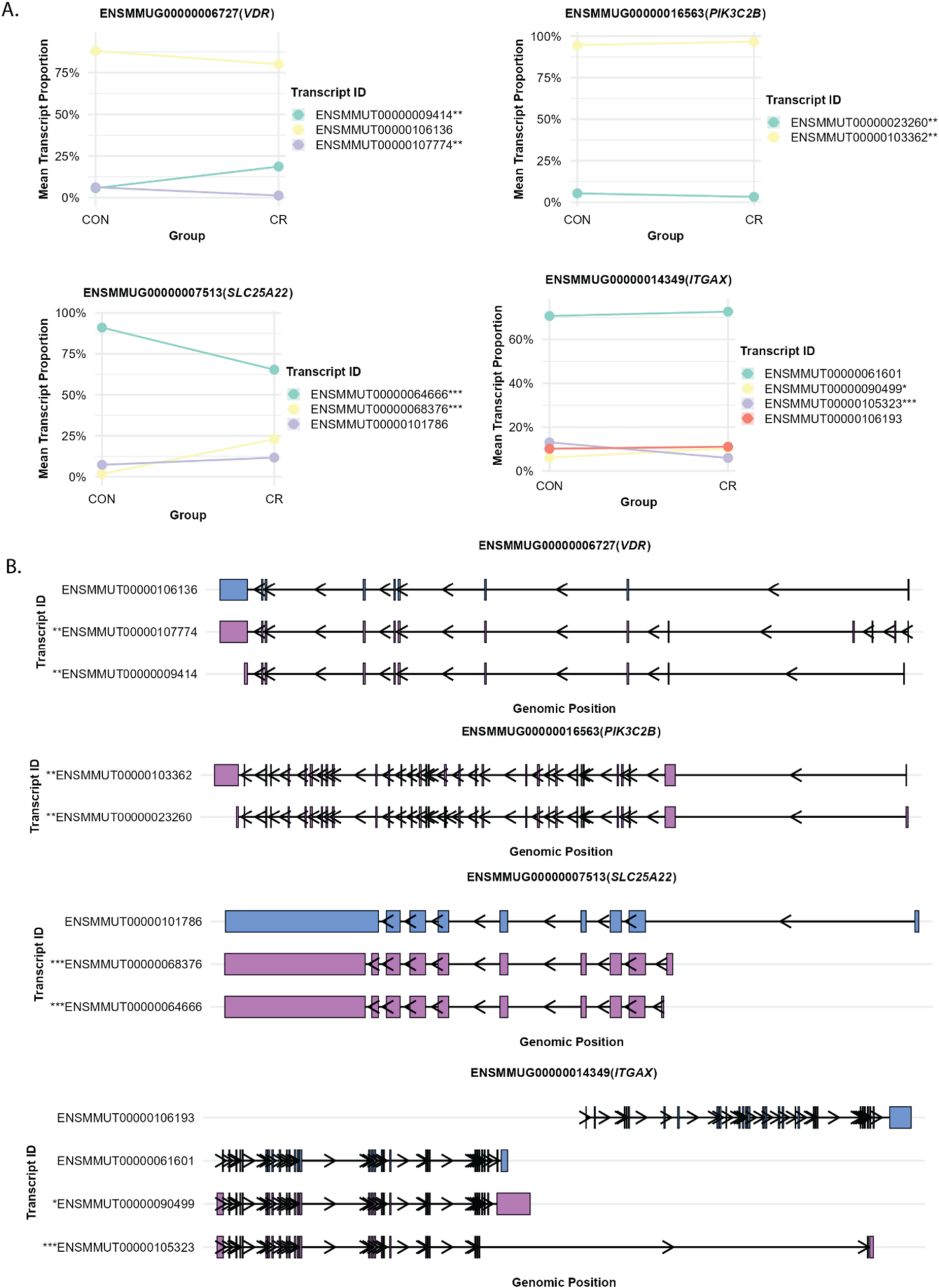
Figure shows four genes (ITGAX, SLC25A22, VDR, PIK3C2B) and associated splicing variants and their transcript structures. **A** Mean transcript proportions for control (CON) and caloric restriction (CR) groups, with genes on the Y-axis and proportion values on the X-axis. Statistical significance is indicated by asterisks: *** *p* < 0.001, ** 0.001 < *p* < 0.01, * 0.01 < *p* < 0.05. **B** Detailed transcript structures showing specific splicing variants and exon-intron junctions for each gene. Light purple indicates significant splicing variants (*p* < 0.05), while light blue represents non-significant variants

We first focused on splicing variants originating from differentially expressed gene analysis with *p* < 0.01, which revealed 24 splicing variants transcribed from 15 genes (Table S4). Some of these genes, which are known to play roles in CR and muscle physiology, showed marked difference in their mean transcript proportion, with each highlighted gene exhibiting exactly two splicing variants that displayed reciprocal mean transcript proportions between the CR and control groups (Fig. 4). *VDR* (Vitamin D Receptor) produced two significant splicing variants (ENSMMUT00000107774, ENSMMUT00000009414). *VDR* is essential for muscle function and mitochondrial activity [116], and since caloric restriction influences systemic vitamin D metabolism [55], these variants may modulate VDR-dependent signaling pathways critical for CR adaptation. *PIK3C2B* (Phosphatidylinositol-4-Phosphate 3-Kinase Catalytic Subunit Type 2 Beta) produced two significant splicing variants (ENSMMUT00000023260, ENSMMUT00000103362). As a key component of the PI3K/AKT pathway that is down-regulated by CR [35], *PIK3C2B* has critical roles in muscle function, aging, and sarcopenia [117]. *SLC25A22* (Solute carrier family 25 member 22) encodes a mitochondrial glutamate.

Transporter (GC1) essential for glutamate import into mitochondria and energy production [118]. *SLC25A22* also produce two significant splicing variants (ENSMMUT00000068376, ENSMMUT00000064666). Given its role in mitochondrial energy metabolism, *SLC25A22* may influence muscle function and aging pathways, especially under the metabolic stress induced by CR. *ITGAX* (significant variant ENSMMUT00000105323): Integrin alpha X is associated with Integrin signaling and immune cell activation, CR has well documented anti-inflammation effect and reduces the expression of many inflammatory markers, including CD11c [119].

We next examined the genes corresponding to the top 30 most significant splicing variants (Table S5). In total, 29 genes were associated with at least one highly significant splicing variant (*p* < 0.01). Among these, we already discussed two genes *SLC25A22* and *ITGAX*, besides five other genes demonstrated particularly notable associations with core CR, muscle function, and aging pathways. *EPRS1* (significant variant ENSMMUT00000045292): Glutamyl-prolyl-tRNA synthetase 1 is associated with core CR signaling through the mTOR/AKT pathway [120]. *PPP2R2A* (Protein Phosphatase 2 Regulatory Subunit Balpha) gene with significant variant ENSMMUT00000055302: This PP2A regulatory subunit B55α affects AKT and downstream signaling cascades involved in muscle growth/atrophy and metabolic regulation [121, 122]). IFI16 (variant ENSMMUT00000006363): Interferon gamma-inducible protein 16 plays pivotal roles in innate immune responses [123, 124], which are increasingly recognized as central to aging and CR-mediated health benefits. *PPP5C* (Protein phosphatase 5 catalytic) with significant variant ENSMMUT00000000327: *PPP5C* subunit regulates circadian clock function and cellular stress responses [125, 126], both critical components of CR adaptation and healthy aging. *SERAC1* (Serine Active Site Containing 1) with significant variant ENSMMUT00000077020: *SERAC1* is involved in mitochondrial phospholipid metabolism, a hallmark of aging mitigated by CR.

## Conclusion

Our study align with previous research in model organisms and our previous human studies [18], reinforcing the role of CR in activating molecular pathways linked to skeletal muscle health, longevity, and healthy aging. By examining skeletal muscle transcriptomes in NHPs, this study advances our understanding of CR’s impact at the molecular level. We identified both new and previously described molecular signatures that together highlight a systemic reprogramming of cellular pathways in response to chronic CR. Specifically, the upregulation of genes linked to mitochondrial biogenesis and oxidative phosphorylation, indicating improved energy production efficiency and metabolic flexibility. Elevated expression of genes related to lipid metabolism suggests a shift toward using lipids for energy, likely to help to improve energy efficiency and decrease reliance on glycolytic pathways. At the same time, the enrichment of neuronal signaling and synaptic plasticity pathways hints at a possible neuroprotective role of CR, which may have important implications for maintaining neuromuscular function during aging.

On the other hand, we noted the downregulation of pathways involved in inflammatory signaling, immune activation, extracellular matrix (ECM) remodeling, and ribosomal RNA processing. These changes indicate a coordinated decrease in anabolic and pro-inflammatory activities, supporting a state of cellular maintenance and lowering metabolic stress. The suppression of ECM-related genes may also indicate reduced tissue turnover or fibrosis, helping to preserve muscle architecture over time.

Analysis of splicing variants revealed that multiple genes produce more than one transcript, highlighting regulatory hotspots for alternative splicing. Several of these variants occur in genes directly linked to muscle function, metabolic regulation, and aging. Overall, these alternatively spliced genes converge on pathways related to mitochondrial adaptation, immune signaling, chromatin remodeling, and muscle metabolism, suggesting that alternative splicing serves as a key integrative mechanism fine-tuning CR-responsive pathways in skeletal muscle.

Despite these strengths, several limitations should be acknowledged. The findings are based solely on transcriptomic data and were not independently validated by qPCR or protein-level assays because no tissue was available for such validation. Additionally, the study was conducted in a single non-human primate cohort, and replication in independent cohorts would strengthen generalizability. As functional assays were not performed, the biological consequences of the identified pathways and splicing events cannot be directly inferred.

These transcriptomic shifts align with established CR-induced changes in rodents and other model organisms, reinforcing the evolutionary conservation of CR’s impact on aging biology. Importantly, our findings extend these observations to an NHP model, which shares closer physiological and aging characteristics with humans. Collectively, the data supports the hypothesis that CR promotes a muscle phenotype characterized by enhanced metabolic health, reduced cellular stress, and improved resilience, all of which are hallmarks of extended health span. These insights may inform translational strategies aimed at mimicking the benefits of CR in human aging and metabolic disease.

## Supplementary Information

Supplementary Material 1.

Supplementary Material 2.

## Data Availability

The datasets generated and analyzed during the current study are available in the NCBI Gene Expression Omnibus (GEO) under accession number GSE316563 ( https:/www.ncbi.nlm.nih.gov/geo/query/acc.cgi? acc=GSE316563 ). All materials supporting the findings of this study are provided within the manuscript and supplementary information. Additional details required to reproduce or reanalyze the results are available from the lead contact upon reasonable request.

## Abbreviations

NHP: Non-human primates
CR: Calorie restriction
CON: Control
ECM: Extracellular matrix
TCA: Citric acid
