# Supplementary material for: Caloric restriction reprograms skeletal muscle molecular pathways in non-human primates: potential relevance to human aging biology
Source: Skelet Muscle. 2026 May 8;16:22. doi: 10.1186/s13395-026-00422-9 (PMC13237944; doi:10.1186/s13395-026-00422-9)
Supplement: Supplementary file 2 — Supplementary Material 2. [file 13395_2026_422_MOESM2_ESM.docx]

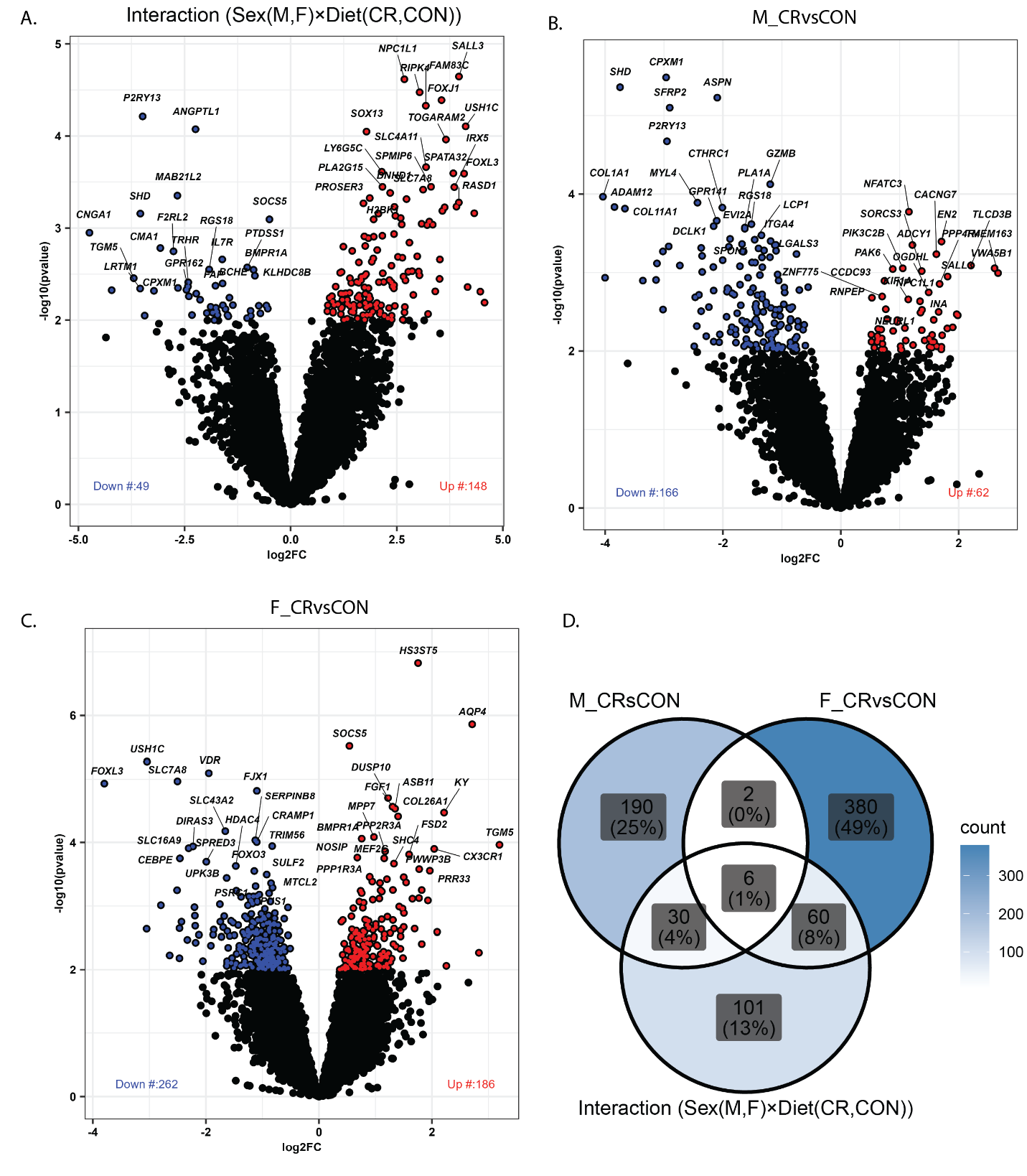


**Figure S1. Differential gene expression analysis reveals sex-specific transcriptional responses to caloric restriction (CR). Volcano plots display log2 fold change (log2FC) versus statistical significance (−log10 p-value) for all detected transcripts. Red points indicate significantly upregulated genes in CR (log2FC > 0, p < 0.01), blue points indicate significantly downregulated genes in CR (log2FC < 0, p < 0.01), and gray points represent non-significant genes. The numbers shown in each panel indicate the total number of significantly upregulated (red) and downregulated (blue) genes (p < 0.01).**(A) Analysis of the sex (male, female) × diet (CR, CON) interaction, adjusted for age, weight, and batch. (B) Sex-stratified analysis in males, adjusted for age, weight, and batch. (C) Sex-stratified analysis in females, adjusted for age, weight, and batch.
(D) Venn diagram showing the overlap of significantly differentially expressed genes (p < 0.01) among the interaction analysis and the male- and female-specific analyses.
